# Sodium Metabisulfite in Food and Biological Samples: A Rapid and Ultra-Sensitive Electrochemical Detection Method

**DOI:** 10.3390/mi13101707

**Published:** 2022-10-10

**Authors:** Ruxandra-Maria Ilie-Mihai, Bianca Cristina Ion, Jacobus (Koos) Frederick van Staden

**Affiliations:** Laboratory of Electrochemistry and PATLAB, National Institute of Research for Electrochemistry and Condensed Matter, 202 Splaiul Independentei Str., 060021 Bucharest, Romania

**Keywords:** sodium metabisulfite, square wave voltammetry, food samples, electrochemical sensor

## Abstract

The primary benefit of using sulfites as a food additive is their antimicrobial and antioxidant properties, which stop fungi and bacteria from growing in a variety of foods. The application of analytical methods is necessary to ensure food quality control related to the presence of sulfites in a variety of foods. For the detection of sodium metabisulfite in food and urine samples, two sensors based on reduced graphene oxide doped with Pd paste and modified with 5,10,15,20-tetraphenyl-21H,23H-porphyrin and 5,10,15,20-tetrakis (pentafluorophenyl chloride)-21H,23H-iron (III) porphyrin were proposed. The new sensors were evaluated and characterized using square wave voltammetry. The response characteristics showed that the detection limits for the sensors were 3.0 × 10^−12^ mol L^−1^ for TPP/rGO@Pd0 based sensors and 3.0 × 10^−11^ mol L^−1^ for Fe(TPFPP)Cl/rGO@Pd0 based sensors while the quantification limits were 1.0 × 10^−11^ mol L^−1^ for TPP/rGO@Pd0 based sensors and 1.0 × 10^−10^ mol L^−1^ for Fe(TPFPP)Cl/rGO@Pd0 based sensors. The sensors can be used to determine sodium metabisulfite in a concentration range between 1.0 × 10^−11^ and 1.0 × 10^−7^ mol L^−1^ for TPP/rGO@Pd0 based sensors and between 1.0 × 10^−10^ mol L^−1^ and 1.0 × 10^−6^ mol L^−1^ for Fe(TPFPP)Cl/rGO@Pd0 based sensors. A comparison between the proposed methods’ results and other analytical applications is also presented.

## 1. Introduction

Food additives have become increasingly important in the modern food industry as a result of changes in human lifestyle and subsequent nutritional demands [1,2]. It is common practice to use food preservatives when storing food for extended periods of time to prevent spoilage due to microbial or fungal growth or unintended chemical changes [3]. It is unclear what toxic effects and mechanisms of action these chemicals have despite their widespread use in the food industry. New research suggests a link between excessive consumption of certain food additives and an array of human diseases [4]. Concerns about food safety and the possible dangers of food additives have grown in response to this issue over the years. Sodium metabisulfite (SMB), a common preservative in the food industry, was found to have neurotoxic effects and increase tissue damage indicators in in vivo studies with animals [5].

In many food products, particularly fruits, vegetables, seafood, pastry, and alcoholic beverages, SMB, also known as sodium pyrosulfite (Na_2_S_2_O_5_), is a synthetic food additive that is used as an antioxidant and antibacterial preservative [6,7]. Additionally, it is utilized as an excipient in the pharmaceutical sector to increase the stability of active principles [8]. High oral doses of SMB have been shown to increase cell damage indices and may have genotoxic effects on mouse tissues according to previous reports [9]. Furthermore, additional in-vivo studies showed that high levels of SMB can cause apoptosis and lipid peroxidation in rat stomach tissue [10]. Numerous fields, including pharmacology, environmental analysis, food sciences, enzymatic kinetics, and medical diagnostics, have benefited from the use of electrochemistry-based analytical methods and sensors [11,12,13]. The creation of electrochemical sensors based on nanomaterials is an active research area that is anticipated to produce cutting-edge technologies for maintaining food integrity that will displace current methods. Reduced graphene oxide (rGO) is one unique material that can be utilized for the creation of electroanalytical sensors. Similar to graphene nanosheets, rGO is composed of a few layers of sp^2^ carbon atoms and exhibits a singular two-dimensional structure. This pi-conjugated system results in high electron mobility and quick charge transfer, which makes an electro-catalytic effect visible [14]. According to studies [15,16], graphene-based electrodes are more electrocatalytically active and conductive than other carbon-based materials. Numerous studies [17] have shown that adding noble metal nanoparticles to graphene can improve the electrocatalytic activity of the material [18,19,20]. Because of their excellent electrocatalytic properties, palladium (Pd) nanoparticles in particular have been extensively investigated for the determination of various analytes [21]. They have superior optical qualities and larger surface energy, which leads to their aggregation and lowers their catalytic efficiency. In order to improve their catalytic efficiency, many optimizations in terms of their structure and crystal characteristics are researched in the literature. For chemical reactions, particularly those involving electron transfer, porphyrins [22,23] and metalloporphyrins are well known for their electrocatalytic properties [24,25].

As a result, the current study demonstrates the development and validation of a straightforward method for the quantification of sodium metabisulfite in foods and biological samples. This method was demonstrated in cookies, bean flakes, horseradish paste, and urine samples as proof of concept.

## 2. Materials and Methods

Sodium metabisulfite, 5,10,15,20-tetraphenyl-21H,23H-porphyrin (TPP), 5,10,15,20-tetrakis(pentafluorophenyl)-21H,23H-porphyrin iron(III) chloride (Fe(TPFPP)Cl), monosodium phosphate, disodium phosphate, acesulfame K, sucrose, glucose, sodium benzoate, d-sorbitol, iron sulphate heptahydrate, maltodextrin, sodium nitrate, and ammonium chloride were purchased from Sigma-Aldrich, and paraffin oil (d_4_^20^, 0.86 g cm^−1^) was purchased from Fluka (Buchs, Sweden). Reduced graphene oxide dopped with Pd(0) (rGO@Pd0) was acquired from NanoInnova Technologies. The phosphate buffer solution (PBS, 0.1 mol L^−1^) was prepared by mixing monosodium phosphate and disodium phosphate solutions. The pH of the buffer solution was adjusted using different amounts of 0.1 mol L^−1^ NaOH or HCl solutions to obtain different pH values (2.0, 2.5, 3.0, 3.5, 4.0, 4.5, 5.0, 5.5, 6.0, 7.0, and 8.0). The stock solution of 1.0 × 10^−2^ mol L^−1^ sodium metabisulfite was prepared fresh before measurements in deionized water.

Using a mini potentiostat EmSTAT Pico (software PsTrace 5.9 PalmSens) connected to a laptop for data acquisition, cyclic voltammetry (CV), square wave voltammetry (SWV), and electrochemical impedance spectroscopy (EIS) measurements were performed.

**Scheme 1:**
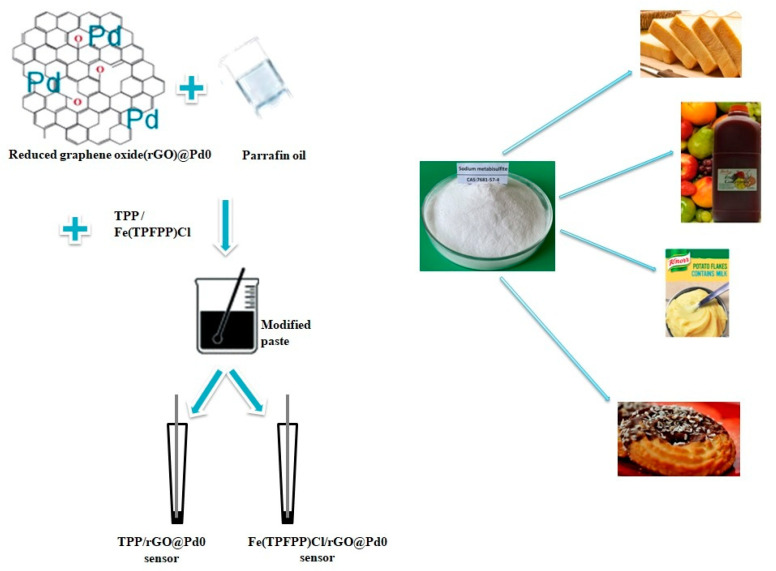
Design of the modified electrochemical sensors.

All electrochemical experiments were conducted at room temperature. The modified nanographene paste electrode Ag/AgCl (0.1 mol L^−1^ KCl) and Pt-wire were used as the working reference and auxiliary electrodes, respectively, and immersed in an electrochemical cell for recording the results. A Mettler Toledo pH meter was used to adjust the pH. An amount of 100 mg of palladium-doped reduced graphene oxide was mixed with paraffin oil until a homogeneous paste was obtained. The modified paste (Scheme 1) was prepared by the addition of 25 μL from a solution of 5,10,15,20-tetraphenyl-21H,23H-porphyrin (1.0 × 10^−3^ mol L^−1^ in tetrahydrofuran) and 25 μL from a solution of 5,10,15,20-tetrakis(pentafluorophenyl)-21H,23H-porphyrin iron(III) chloride (1.0 × 10^−3^ mol L^−1^ in tetrahydrofuran) to the bare paste. The TPP/rGO@Pd0 and Fe(TPFPP)Cl/rGO@Pd0 sensors were obtained, respectively, by placing the paste into non-conducting plastic tubes (with an internal diameter of 25 μm) in which a silver wire inserted into the paste served as an electrical contact between the paste and an external circuit.

The newly developed sensors were used to detect Na_2_S_2_O_5_ in three types of food samples (cookie, bean flakes, and horseradish paste) bought from a Romanian supermarket and also a biological sample obtained from a healthy volunteer. The samples were diluted in PBS pH = 4.5, in a 1:1 (*v*/*v*) ratio, and afterward spiked with different concentrations of sodium metabisulfite.

## 3. Results

### 3.1. Electrochemical Characterization of the Sensors

**Figure 1:**
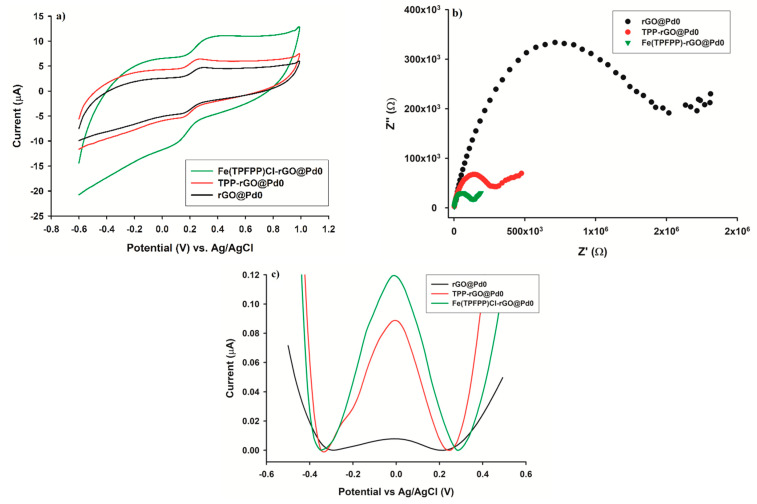
(**a**) Cyclic voltammograms of the current plotted against the potential (working conditions: step potential 0.025 V and scan rate 0.1 V s^−^^1^) in a solution of 5.0 × 10^−^^3^ mol L^−^^1^ K_3_[Fe(CN)_6_] (0.1 mol L^−^^1^ KCl) using the rGO@Pd0 (black line), TPP/rGO@Pd0 (red line), and Fe(TPFPP)Cl/rGO@Pd0 (green line). (**b**) Electrochemical impedance spectra recorded for rGO@Pd0 (black line), TPP/rGO@Pd0 (red line), and Fe(TPFPP)Cl/rGO@Pd0 (green line) in a solution of 5.0 × 10^−^^3^ mol L^−^^1^ K_3_[Fe(CN)_6_] (0.1 mol L^−^^1^ KCl (conditions: frequency range between 1.0 × 10^5^ and 1.0 × 10^−^^1^ Hz). Inset: equivalent circuit diagram of the electrochemical interface used to fit the impedance spectra where Rs is the solution resistance, CPE is the constant phase element, Rct is the electron-transfer resistance, and W is the Warburg diffusion resistance. (**c**) The recorded square wave voltammograms in PBS pH 4.5 containing 1.0 × 10^−^^7^ mol L^−^^1^ Na_2_S_2_O_5_ for rGO@Pd0 (black line), TPP/rGO@Pd0 (red line) and Fe(TPFPP)Cl/rGO@Pd0 (green line); working conditions: step potential, 0.03 V; amplitude, 0.1 V; and frequency, 10 Hz.

CV, EIS, and SWV were used as the characterization techniques for the bare sensor, the simple reduced graphene oxide dopped with palladium (rGO@Pd), and the two modified sensors: reduced graphene oxide dopped with palladium based on 5,10,15,20-tetraphenyl-21H,23H-porphyrin (TPP/rGO@Pd0) and based on 5,10,15,20-tetrakis(pentafluorophenyl)-21H,23H-porphyrin iron(III) chloride (Fe(TPFPP)Cl/rGO@Pd0). To analyze the chemical response of the sensors, cyclic voltammetry was used (Figure 1). The CVs (Figure 1a) were conducted using the rGO@Pd0, TPP/rGO@Pd0, and Fe(TPFPP)Cl/rGO@Pd0 as working electrodes in a solution of 5.0 × 10^−3^ mol L^−1^ K_3_[Fe(CN)_6_] (0.1 mol L^−1^ KCl) at potentials between −0.6 V and 1.0 V. It is evident that the conductivity of the sensor increased after the rGO electrode was modified with TPP and Fe(TPFPP)Cl. One of the reasons for the increased conductivity is the given function of porphyrins as a modifier. Due to their unique structure, porphyrins can have the ability to control redox reactions that the target analytes mediate. This has therefore shown that the modification was made, and that the electrochemical response was enhanced. The EIS study was carried out in order to investigate the interface of the sensors over a frequency spectrum ranging from 1.0 × 10^5^ to 1.0 × 10^−1^ Hz. Every single one of the EIS measurements was carried out in a solution that contained 5.0 × 10^−3^ mol L^−1^ K_3_[Fe(CN)_6_] (0.1 mol L^−1^ KCl). Figure 1b depicts Nyquist diagrams. Figure 1b demonstrates that at low frequencies, where charge-transfer resistance is high (Rct = 2.61 × 10^5^ Ω), rGO@Pd0 exhibited a large, well-defined semicircle. The diameter of the semicircle shrank (Rct = 7.16 × 10^4^ Ω) after rGO@Pd0 was modified with TPP. The bare sensor that had been modified with Fe(TPFPP)Cl exhibited even a smaller semi-circle (Rct = 1.71 × 10^4^ Ω) afterward. Rct is the charge-transfer resistance obtained from the fitted Nquist plots (Ω) and represents the electrode resistance, being related to the surface area and the electrode conductivity. In conclusion, compared to the unmodified graphene paste electrode, the sensors that had been treated with TPP and Fe(TPFPP)Cl exhibited a smaller semicircle and higher Rct values. The EIS results for a 5.0 × 10^−3^ mol/L K_3_[Fe(CN)_6_] (0.1 mol L^−1^ KCl) solution showed good agreement with the CV results. To compare the electrochemical behavior between the three electrodes, the bare sensor and the two modified sensors, a solution containing 1.0 × 10^−7^ mol L^−1^ sodium metabisulfite buffered with PBS at pH 4.5 was analyzed using an SWV method. From Figure 1c, it can be seen that among the three sensors, the Fe(TPFPP)Cl/rGO@Pd0 and TPP/rGO@Pd0 sensors gave the best results for sodium metabisulfite oxidation. After further characterization and testing, the two sensors were evaluated for their ability to electrochemically determine the presence of Na_2_S_2_O_5_ in samples of cookies, potato flakes, horseradish paste, and urine.

**Figure 2:**
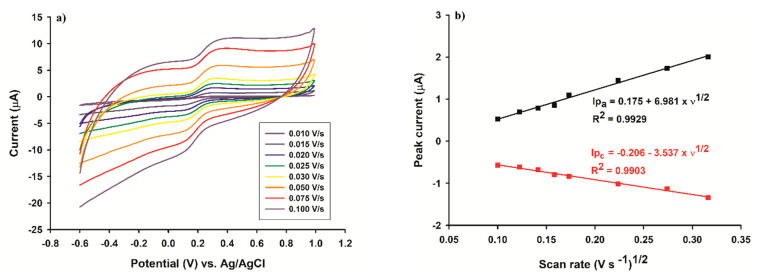
(**a**) Cyclic voltammograms in a solution of 5.0 × 10^−^^3^ mol L^−^^1^ K_3_[Fe(CN)_6_] (0.1 mol L^−^^1^ KCl) at different scan rates from 0.010 to 0.100 V s^−^^1^ using the Fe(TPFPP)Cl/rGO@Pd0 sensor (working conditions: potential range from −0.6 to 1.0 V and step potential 0.025 V). (**b**) Dependence of the peak current on the square root of the scan rate using the Fe(TPFPP)Cl/rGO@Pd0 sensor.

**Figure 3:**
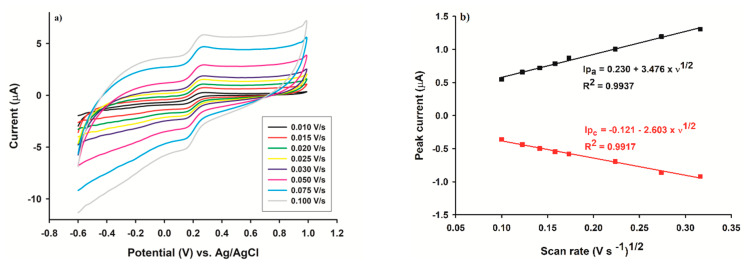
(**a**) Cyclic voltammograms in a solution of 5.0 × 10^−^^3^ mol L^−^^1^ K_3_[Fe(CN)_6_] (0.1 mol L^−^^1^ KCl) at different scan rates from 0.010 to 0.100 V s^−^^1^ using the TPP/rGO@Pd0 sensor (working conditions: potential range from −0.6 to 1.0 V and step potential 0.025 V). (**b**) Dependence of the peak current on the square root of the scan rate using the TPP/rGO@Pd0 sensor.

Calculating the electroactive surface area of the four sensors was done with the help of the Randles–Sevcik equation [26] for quasi-reversible processes. This allowed for the investigation of the electrocatalytic activity of the sensors. The equation for the peak current intensity can be summarized as follows:

$$Ip_{a}=\left(2.69\times 10^{5\;}\right)\;n^{3/2}\;AC_{0}D_{R}^{1/2}\upsilon^{1/2}$$

where: $Ip_{a}$—anodic peak current (A), n—number of transferred electrons (in this instance, n = 1), A—active surface area of the electrode (cm^2^), C_0_—concentration of K_3_[Fe(CN)_6_] (mol cm^−3^), D_R_—diffusion coefficient (7.60 × 10^−6^ cm^2^ s^−1^), and υ—scan rate (V s^−1^). The experiment was carried out in a solution that contained 5.0 × 10^−3^ mol L^−1^ of K_3_[Fe(CN)_6_] and 0.1 mol L^−1^ of KCl. The fact that the anodic and cathodic peaks $Ip_{a}$ and $Ip_{c}$ showed a linear dependency on the square root of the scan rate (Figure 2 and Figure 3) despite the fact that the scan rate varied from 0.010 to 0.100 V s^−1^ provides evidence that the redox process was controlled by diffusion. Figure 2a and Figure 3a depict the pattern that emerges as the scan rate and current intensity both continue to increase over time. Figure 2b and Figure 3b depict the linear dependences of the two peaks $Ip_{a}$ vs. υ^1/2^ and $Ip_{c}$ vs. υ^1/2^, respectively. In comparison, the sensor based on Fe(TPFPP)Cl/rGO@Pd0 presents the highest active area (0.019 cm^2^) beside the other modified sensor TPP/rGO@Pd0 (0.0095 cm^2^) and compared to the unmodified sensor rGO@Pd0 (0.0009 cm^2^).

### 3.2. The Influence of the pH Value on the Electrochemical Behavior of the Sensors

**Figure 4:**
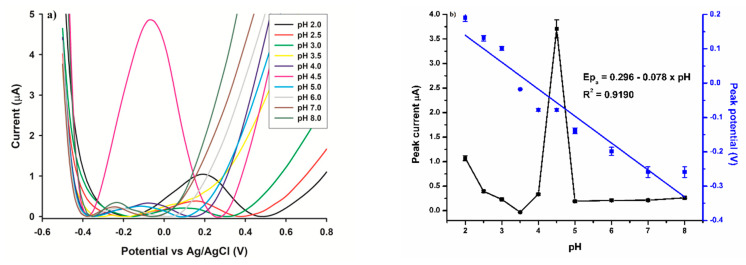
(**a**) Square wave voltammograms of 1.0 × 10^−^^4^ mol L^−^^1^ Na_2_S_2_O_5_ using the TPP/rGO@Pd0 sensor in PBS at pH values 2.0−8.0. (**b**) The effect of the pH on the peak current (black dots) and the linear dependence of the peak potential with the pH (blue line).

**Figure 5:**
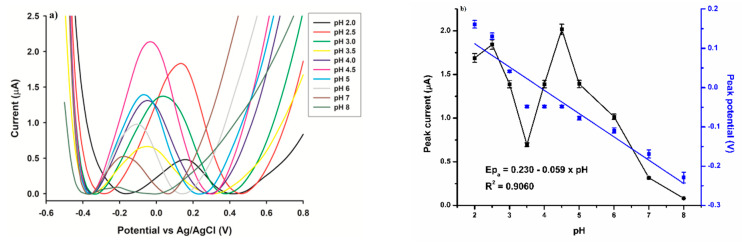
(**a**) Square wave voltammograms of 1.0 × 10^−^^4^ mol L^−^^1^ Na_2_S_2_O_5_ using the Fe(TPFPP)Cl/rGO@Pd0 sensor in PBS at pH values 2.0−8.0. (**b**) The effect of the pH on the peak current (black dots) and the linear dependence of the peak potential with the pH (blue line).

When conducting electrochemical measurements (Figure 4a and Figure 5a), it is absolutely necessary to take note of the pH level of the solution; therefore, a 100 μmol L^−1^ sodium metabisulfite solution was buffered with PBS with different pH values (2.0, 2.5, 3.0, 3.5, 4.0, 4.5, 5.0, 5.5, 6.0, 7.0, and 8.0). From Figure 4b, it can be seen that in the case of the TPP/rGO@Pd0 sensor, there is a slight current increase at around pH 2.0, followed by a decrease to pH 3.5, reaching a maximum value in the current at pH 4.5, succeeded by a plateau from pH = 5–8. When it comes to the Fe(TPFPP)Cl/rGO@Pd0 sensor (Figure 5b), one can see that at pH = 2.5 a high increase in the current is obtained, followed by the decline in the current value. At pH = 4.5 a maximum current value is achieved, continued by a final reduction in current value to pH = 8. As demonstrated in both figures, the highest current intensity of sodium metabisulfite was observed when the pH was 4.5 and when both sensors were used. The correlation between the pH values and the peak potential (Epa) is shown in Figure 4b and Figure 5b. Good observation can be made regarding the equation from Figure 4b when the TPP/rGO@Pd0 sensor was used; it can be seen that the obtained slope value is −0.078 V pH^−1^ and close to the Nernstian theoretical value of 0.059 V pH^−1^ which states that the number of protons involved in the oxidation process is equal to the number of electrons that are present in the process. On the other hand, Figure 5b provides an interesting result from the equation, Epa (V) = 0.230−0.059 pH, with a regression coefficient (R^2^) of 0.9060 obtained for Fe(TPFPP)Cl/rGO@Pd0, where the slope value of 0.059 V pH^−1^ is the same as the Nernstian theoretical value of 0.059 V pH^−1^, thus proving that the number of protons and electrons involved in the oxidation process are equal. By dissolving sodium metabisulfite in water, a pH of 4.5 was observed. At lower or higher pH values, sodium metabisulfite decomposes. This observation is correlated with the highest peak obtained at this pH value.

### 3.3. Response Characteristics of the Electrochemical Sensors

**Figure 6:**
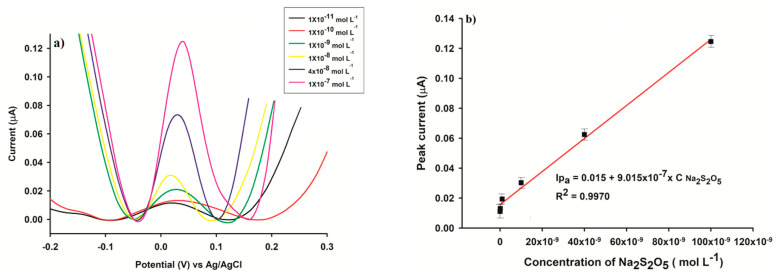
(**a**) Square wave voltammograms recorded with the TPP/rGO@Pd0 sensor in PBS pH 4.5 containing different concentrations of Na_2_S_2_O_5_ from 1.0 × 10^−^^11^ mol L^−^^1^ to 1.0 × 10^−^^7^ mol L^−^^1^. (**b**) Calibration curve obtained with the TPP/rGO@Pd0 sensor from 1.0 × 10^−^^11^ mol L^−^^1^ to 1.0 × 10^−^^7^ mol L^−1^. Working conditions: step potential, 0.03 V; amplitude, 0.1 V; and frequency, 10 Hz.

At the ideal pH level (pH = 4.5), the response characteristics of the proposed electrochemical sensors were identified using square wave voltammetry and are listed in Table 1. A wide concentration range, high sensitivities, and low limits of quantification and determination for the proposed sensors were obtained thanks to the optimal working conditions and the electrocatalytic capacity of the porphyrins used in their design. The calibration graph for sodium metabisulfite is shown in Figure 6b along with the peaks that were obtained during the calibration of the TPP/rGO@Pd0 sensor. The linear concentration range was from 1.0 × 10^−11^ mol L^−1^ to 1.0 × 10^−7^ mol L^−1^ with a correlation coefficient of 0.9970. The LOD and LOQ were calculated to be 3.0 × 10^−12^ and 1.0 × 10^−11^ mol L^−1^, respectively. The values of LOD and LOQ are as follows: LOD = 3 s/m and LOQ = 10 s/m; where s is the standard deviation of the peak current of the blank (4 measurements) and m represents the slope of the calibration curve. The sensitivity of the TPP/rGO@Pd0 sensor was 9.015 × 10^−7^ μA/mol L^−1^.

The inorganic salt of sulfuric acid sodium metabisulfite (Na_2_S_2_O_5_) dissolves in water to produce sodium, bisulfite, and sulfite ions [27]: Na_2_S_2_O_5_ + H_2_O = 2Na+ + 2HSO^3−^ and HSO^3−^ = H^+^ + SO_3_^2−^. Bisulfite anions are changed into sulfate anions when oxygen is present: 2HSO^3-^ + O_2_ = 2H+ + 2SO_4_^2−^.

**Figure 7:**
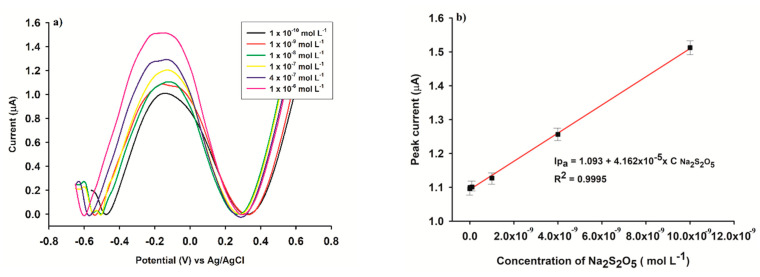
(**a**) Square wave voltammograms recorded with the Fe(TPFPP)Cl/rGO@Pd0 sensor in PBS pH 4.5 containing different concentrations of Na_2_S_2_O_5_ from 1.0 × 10^−^^10^ mol L^−1^ to 1.0 × 10^−^^6^ mol L^−^^1^. (**b**) Calibration curve obtained with the Fe(TPFPP)Cl/rGO@Pd0 sensor from 1.0 × 10^−^^10^ mol L^−^^1^ to 1.0 × 10^−^^6^ mol L^−^^1^. Working conditions: step potential, 0.03 V; amplitude, 0.1 V; and frequency, 10 Hz.

Regarding the Fe(TPFPP)Cl/rGO@Pd0 sensor, Figure 7b presents the peaks acquired after calibration measurements in addition to the calibration graph of sodium metabisulfite. For the LOD and LOQ calculations, the above-mentioned formulas were used. Therefore, very good results were obtained: linear concentrations from 1.0 × 10^−10^ mol L^−1^ to 1.0 × 10^−6^ mol L^−1^, LOD and LOQ values of 3.0 × 10^−11^ mol L^−1^ and 1.0 × 10^−10^ mol L^−1^, a far superior correlation coefficient of 0.9995, and a sensitivity of 4.162 × 10^−5^ μA/mol L^−1^.

### 3.4. Interference Studies of the Electrochemical Sensors

In order to test for potential interferences in the detection of sodium metabisulfite, a number of ions, including NH_4_^+^, Fe^2+^, Na^+^, and organic species, including acesulfame K, sucrose, glucose, sodium benzoate, d-sorbitol, and maltodextrin, were tested. The substances frequently found with sodium metabisulfite in cookies, bean flakes, horseradish paste, and urine samples were chosen as potentially interfering substances. The maximum interference concentration that resulted in a change in current intensity in terms of relative error (5% acceptance level), bias (%), and signal change (%) were referred to as the tolerance limit. All measurements were conducted using sodium metabisulfite solutions (1.0 × 10^−8^ mol L^−1^) buffered with PBS at a pH of 4.5. The experimental results when the TPP/rGO@Pd0 sensor was used, exhibited no influence on the detection of sodium metabisulfite when an excess of 10-fold of NH^4−^ ion, 25-fold of sodium benzoate, 50-fold of glucose and sucrose, and 100-fold of Fe^2+^, Na^+^ ions, acesulfame K, d-sorbitol and maltodextrin was added (Table 2), which indicated that the proposed sensor presented a good selectivity on the determination of Na_2_S_2_O_5_.

In the case of the second sensor, the Fe(TPFPP)Cl/rGO@Pd0 sensor, the results in Table 3 show that an added excess of 10-fold of NH_4_^+^, Fe^2+^, Na^+^ ions and glucose, 25-fold of sucrose, 50-fold of sodium benzoate, and 100-fold of acesulfame K, d-sorbitol and maltodextrin manifests no influence on the detection of sodium metabisulfite, also proving that the sensor has a good selectivity on the determination of Na_2_S_2_O_5_. Since porphyrins and metal complexes (including Fe(III)) with porphyrins mimic the enzymes’ activity, it follows that they selectively promote the oxidation of sodium metabisulfite. The artificial enzymes TPP and Fe(TPFPP)Cl are acting as electrocatalysts in a process that is not a substrate-binding reaction. Since porphyrins are enzyme mimics, their use as artificial enzymes in electrochemistry has been thoroughly investigated [28,29,30,31].

### 3.5. Reproducibility, Repeatability, and Stability

**Figure 8:**
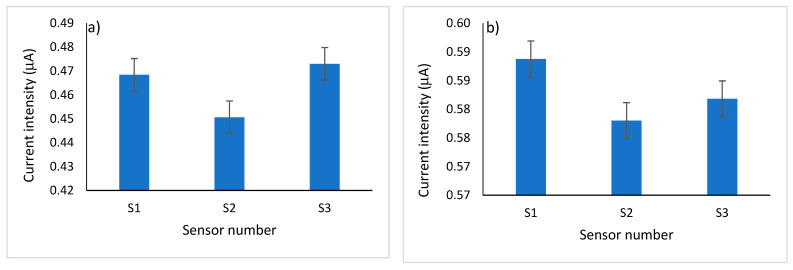
Reproducibility of the (**a**) TPP/rGO@Pd0 and (**b**) Fe(TPFPP)Cl/rGO@Pd0 sensor.

The repeatability, reproducibility, and stability of the developed sensors (TPP/rGO@Pd0 and Fe(TPFPP)Cl/rGO@Pd0) were investigated using a solution of Na_2_S_2_O_5_ (1.0 × 10^−8^ mol L^−1^) in PBS pH 4.5 under the optimal experimental conditions by SWV. The reproducibility was analyzed using three new sensors of each type, which were prepared in the same way (Figure 8a,b). The relative standard deviation (RSD%) was calculated to be 2.55% (n = 3) for the TPP/rGO@Pd0 sensor and 0.94% (n = 3) for the Fe(TPFPP)Cl/rGO@Pd0 sensor.

**Figure 9:**
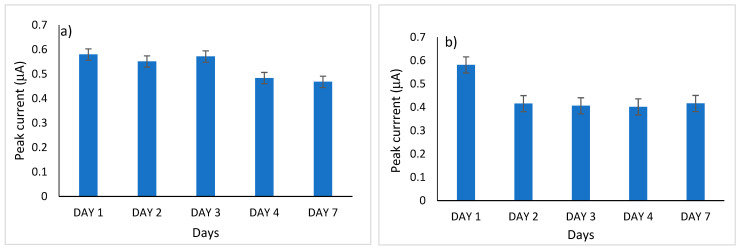
Stability of the sensors for a period of 7 days for: (**a**) TPP/rGO@Pd0 and (**b**) Fe(TPFPP)Cl/rGO@Pd0.

For the within-day repeatability, the repeatability was determined to be 1.54% (n = 5) for the TPP/rGO@Pd0 sensor and 0.97% (n = 5) for the Fe(TPFPP)Cl/rGO@Pd0 sensor. The stability of the sensors was examined for 7 days (Figure 9a,b). The modified electrodes were kept at room temperature throughout the stability examination. After 7 days, in the case of the TPP/rGO@Pd0 sensor, the current intensity of the Na_2_S_2_O_5_ (1.0×10^−8^ mol L^−1^) decreased to a value of 80.70% of the initial value from the first day of assessment; in the case of the Fe(TPFPP)Cl/rGO@Pd0 sensor, the peak current decreased up to a value of 71.59% from the initial value from the first day of assessment. In the case of reproducibility, n = 3 represents the number of sensors. While in the case of repeatability and stability, n = 5 represents the number of determinations.

A comparison between results obtained using the proposed method and those obtained using other analytical methods is shown in Table 4.

### 3.6. Determination of Sodium Metabisulfite in Food and Biological Samples

For the proposed sensors to be used more widely in the monitoring of food quality and the maintenance of food security, they must be validated. The sensors were validated using the standard addition method to show how accurate they are when measuring sodium metabisulfite in cookies, bean flakes, horseradish paste, and urine samples. As can be seen in Table 4 and Table 5, the sodium metabisulfite assay used for the cookies, bean flakes, horseradish paste, and urine samples has a very high degree of reliability.

For sample preparation, ten milliliters of PBS (pH = 4.5) was added to 1 g of cookies and bean flakes. The resulting mixture was vortexed for 4 min and left in the ultrasonic bath for 30 min. One gram of the horseradish paste sample was weighed using an analytical balance, and it was dissolved in 10 mL of PBS (pH = 4.5) and vortexed for 4 min. The urine sample was diluted with PBS pH = 4.5 in a 1:1 (*v*/*v*) ratio and afterward spiked with different concentrations of sodium metabisulfite.

After placing the samples into the electrochemical cell, the peak current was measured. Na_2_S_2_O_5_ concentrations and the obtained values were input into the above calibration equation. Table 4 and Table 5 summarize the recovery, RSD, and bias (%) values.

It can be seen from Table 5 that very good recovery values were obtained when the TPP/rGO@Pd0 sensor was used. For the food samples, the recoveries had high values well above 99%, but with one exception, the spiked cookie sample (10^−8^ mol L^−1^ sodium metabisulfite concentration); however, its recovery values were very close to 99%. Furthermore, the urine sample presented a recovery value above 99.9%. The RSD values for the samples were between 0.05 ± 4.50. In the case of the Fe(TPFPP)Cl/rGO@Pd0 sensor from Table 6, it can be observed that the recoveries from both food and urine samples are above 97% with RSD values ranging from 0.21 to 5.94. Therefore, given the fact that the results presented in Table 5 and Table 6 have very good values, it can be claimed that both sensors, the TPP/rGO@Pd0 and Fe(TPFPP)Cl/rGO@Pd0, demonstrated that they are able to provide an increase in both sensitivity and selectivity of the assay for sodium metabisulfite in food and biological samples.

## 4. Conclusions

Nanomaterials are a promising new tool for improving food safety and quality control monitoring, and their use in technology is sure to pave the way for the creation of even more sensitive electrochemical sensors. Therefore, the present study presents two electrochemical sensors based on reduced graphene oxide doped with Pd paste and modified with 5,10,15,20-tetraphenyl-21H,23H-porphyrin and 5,10,15,20-tetrakis (pentafluorophenyl chloride)-21H,23H-iron (III) porphyrin and were designed, characterized, tested, and validated for the determination of sodium metabisulfite in three different food samples and a biological sample. Both sensors showed very high levels of stability, selectivity, sensitivity, and reproducibility in their measurements. The proposed sensors have the benefit of being able to be utilized in the food industry for the purpose of quality control of food in relation to the determination of the amount of sodium metabisulfite present in samples. In terms of a possible high cost of the device, one should take into consideration that an amount of 10mg of paste can be used for approximately 500 measurements, resulting in a very low price per analysis.

## Figures and Tables

**Table 1:** Response characteristics of the electrochemical sensors used for detection of sodium metabisulfite.

Parameter	Electrochemical Sensors	Parameter
	TPP/rGO@Pd0	Fe(TPFPP)Cl/rGO@Pd0
equation of calibration *	$Ip_{a}$ = 0.015 + 9.015 × 10^−7^ × C_Na2S2O5_, R^2^ = 0.9970	$Ip_{a}$= 1.093 + 4.612 × 10^−5^ × C_Na2S2O5_, R^2^ = 0.9995
linear concentration range (mol L^−1^)	1.0 × 10^−^^11^–1 × 10^−^^7^	1.0 × 10^−^^10^–1 × 10^−^^6^
sensitivity (μA mol L^−1^)	9.02 × 10^−^^7^	4.16 × 10^−^^5^
limit of detection (mol L^−1^)	3.0 × 10^−^^12^	3.0 × 10^−^^11^
limit of quantification (mol L^−1^)	1.0 × 10^−^^11^	1.0 × 10^−^^10^
repeatability of peak current (%, RSD)	2.78	2.75

* <$Ip_{a}$> = μA; <C> = mol L^−1^, n = 10.

**Table 2:** The influence of possible interfering species on the detection of 1.0 × 10^−^^8^ sodium metabisulfite (n = 4) using the TPP/rGO@Pd0 sensor.

Possible Interfering Species	Concentration of Possible Interfering Species (mol L^−1^)	Tolerance Limit	Relative Error (%)	Bias (%)
Fe^2+^	1.0 × 10^−7^	10	0.20	−1.83
Na^+^	1.0 × 10^−7^	10	1.33	−7.33
NH_4_^+^	1.0 × 10^−7^	10	−0.35	0.15
glucose	1.0 × 10^−7^	10	0.74	0.45
sucrose	2.5 × 10^−7^	25	1.50	2.04
sodium benzoate	5.0 × 10^−7^	50	1.10	0.73
acesulfam K	1.0 × 10^−6^	100	−2.25	2.68
d-sorbitol	1.0 × 10^−6^	100	−0.62	1.29
MD	1.0 × 10^−6^	100	−2.41	2.15

**Table 3:** The influence of possible interfering species on the detection of 1.0 × 10^−^^8^ sodium metabisulfite (n = 4) using the Fe(TPFPP)Cl/rGO@Pd0 sensor.

Possible Interfering Species	Concentration of Possible Interfering Species (mol L^−1^)	Tolerance Limit	Relative Error (%)	Bias (%)
NH_4_^+^	1.0 × 10^−7^	10	−1.80	−3.76
sodium benzoate	2.5 × 10^−7^	25	0.35	−0.36
glucose	5.0 × 10^−7^	50	−2.30	2.65
sucrose	5.0 × 10^−7^	50	0.49	−1.74
Fe^2+^	1.0 × 10^−6^	100	−4.68	5.89
Na^+^	1.0 × 10^−6^	100	1.45	−2.35
acesulfam K	1.0 × 10^−6^	100	−1.94	2.37
d-sorbitol	1.0 × 10^−6^	100	−0.83	4.97
MD	1.0 × 10^−6^	100	−0.05	2.03

**Table 4:** Comparison between performances of other analytical methods and the proposed electrochemical method.

Method	Linear Concentration Range (mol L^−1^)	Limit of Detection (mol L^−1^)	Reference
BIA	2.50 × 10^−7^–5.00 × 10^−4^	8.10 × 10^−8^	[32]
RP-HPLC	2.68 × 10^−4^–8.67 × 10^−4^	5.10 × 10^−4^	[33]
CV	1.00 × 10^−4^–1.00 × 10^−3^	1.60 × 10^−4^	[34]
SWV (using TPP/rGO@Pd0)	1.00 × 10^−11^–1.00 × 10^−7^	3.00 × 10^−12^	This work
SWV (using Fe(TPFPP)Cl/rGO@Pd0)	1.00 × 10^−10^–1.00 × 10^−6^	3.00 × 10^−11^	This work

BIA = batch injection analysis; RP-HPLC = reversed-phase high-performance liquid chromatography.

**Table 5:** Determination of sodium metabisulfite in food and biological samples using the TPP/rGO@Pd0 sensor.

Samples	Amount Added (mol L^−1^)	Amount Found (mol L^−1^)	Recovery (%)	RSD (%)	Bias (%)
cookies	-	4.41 × 10^−9^	-	1.30	-
	1.0 × 10^−7^	9.98 × 10^−8^	99.83	0.05	0.17
	1.0 × 10^−8^	9.87 × 10^−9^	98.68	0.24	1.34
bean flakes	-	3.45 × 10^−8^	-	0.47	-
	1.0 × 10^−7^	9.97 × 10^−8^	99.73	0.57	0.27
	1.0 × 10^−8^	1.00 × 10^−8^	99.96	1.21	0.04
horseradish paste	-	6.92 × 10^−8^	-	0.52	-
	1.0 × 10^−7^	9.99 × 10^−8^	99.87	0.80	0.13
	1.0 × 10^−8^	1.00 × 10^−8^	99.97	0.86	0.03
urine	-	-	-	3.78	-
	1.0 × 10^−7^	9.78 × 10^−8^	97.82	4.50	2.23
	1.0 × 10^−8^	9.97 × 10^−9^	99.73	2.16	0.27

**Table 6:** Determination of sodium metabisulfite in food and biological samples using the Fe(TPFPP)Cl/rGO@Pd0 sensor.

Samples	Amount Added (mol L^−1^)	Amount Found (mol L^−1^)	Recovery (%)	RSD (%)	Bias (%)
cookies	-	1.06 × 10^−8^	-	1.31	-
	1.0 × 10^−7^	9.93 × 10^−8^	99.32	2.22	0.67
	1.0 × 10^−8^	9.99 × 10^−9^	99.98	4.48	0.01
bean flakes	-	8.44 × 10^−7^	-	4.03	-
	1.0 × 10^−7^	9.99 × 10^−8^	99.98	1.29	0.01
	1.0 × 10^−8^	9.99 × 10^−9^	99.98	3.45	0.01
horseradish paste	-	9.87 × 10^−9^	-	2.92	-
	1.0 × 10^−7^	9.95 × 10^−8^	99.54	1.86	0.46
	1.0 × 10^−8^	9.71 × 10^−9^	97.11	4.54	2.98
urine	-	-	-	5.94	-
	1.0 × 10^−7^	9.99 × 10^−8^	99.95	0.21	0.05
	1.0 × 10^−8^	9.99 × 10^−9^	99.93	1.04	0.06

## Data Availability

Not applicable.
